# The prevalences of and association between nonmedical prescription opioid use and poor sleep among Chinese high school students

**DOI:** 10.1038/srep30411

**Published:** 2016-07-28

**Authors:** Daiting Tang, Pengsheng Li, Lan Guo, Yan Xu, Xue Gao, Jianxiong Deng, Jinghui Huang, Guoliang Huang, Hong Wu, Yue Yue, Ciyong Lu

**Affiliations:** 1 School of Public Health, Sun Yat-sen University, Department of Medical Statistics and Epidemiology, Guangzhou, 510000, China; 2 Centre for ADR Monitoring of Guangdong, Guangzhou, 510000, China

## Abstract

The purpose of this study was to investigate the prevalences of and association between nonmedical prescription opioid use (NMPOU) and sleep quality among Chinese high school students. A cross-sectional study was conducted in Chongqing high school students in 2012, and questionnaires from 18,686 students were completed and eligible for this study. Demographic and NMPOU information was collected using a self-administered questionnaire. The Chinese Pittsburgh Sleep Quality index (CPSQI) was used to assess the occurrence of poor sleep. Among the total sample, 18.0% were classified as poor sleepers (27.4% of the subjects with past-month NMPOU), and the prevalences of lifetime, past-year and past-month NMPOU were 14.6, 4.6 and 2.8% across the entire sample, respectively. The most commonly used medicine was licorice tablets with morphine (9.1, 2.5 and 1.5% for lifetime, past-year and past-month, respectively), followed by cough syrup with codeine, Percocet, diphenoxylate and tramadol. After adjustment for potential confounders, the association between past-month NMPOU and poor sleep remained significant (AOR = 1.47, 95% CI 1.17 to 1.85). Programs aimed at decreasing NMPOU should also pay attention to sleep quality among adolescents.

The nonmedical use of prescription drugs (including pain medications/opioids, sedatives, tranquillizers and stimulants) has increased dramatically in recent years. In the United States, it has reached epidemic levels^1^. According to the report of the National Youth Risk Behavior Survey (YBRS), 17.8% of students (grades 9–12) surveyed had taken prescription drugs for nonmedical reasons during their lifetime^2^. In China, a province-based survey conducted in 2007–2009 revealed that 6.0% of the students (grades 7–12) had engaged in nonmedical use of four specific prescription drugs during their lifetime^3^. Notably, earlier studies had reported that nonmedical prescription opioid use (NMPOU) is prevalent among adolescents and represents a growing public problem^4^,^5^. The misuse of prescription opioids can lead to numerous problems, such as sexual violence, heroin use, and drug injection^6^, and it causes more deaths than overdoses from heroin and cocaine combined^7^. However, most of these studies were conducted in Western countries, and limited information is available on NMPOU among high school students in developing countries such as China.

Sleep plays a very important role in the physical and mental health of adolescents^8^. One study reported that 39.6% of high school students in southern China suffered sleep disturbances^9^. A number of factors, including demographic characteristics, personal habits and psychosocial characteristics, have been related to sleep quality among adolescents. For example, girls are more likely to report sleep disturbances than boys^10^,^11^, and the prevalence of sleep disturbance varies with age among school-aged children^12^. Among personal habits, smoking has been identified as a risk factor for short sleep duration^13^, and the prevalence of sleep disturbance or insomnia is particularly high among adolescents who drink alcohol^14^,^15^. In addition, several studies have indicated that physical activity influences sleep duration among adolescents^13^,^16^. A study conducted in China identified a moderate association of psychosocial factors, such as depressive symptoms, loneliness and suicide ideation, with poor sleep^9^.

Prior studies have found a bidirectional relationship between sleep and substance abuse, mainly for alcohol, tobacco and illicit drugs^17^,^18^,^19^. More importantly, sleep has further been identified as an effective therapeutic target in adolescents with substance abuse problems, which indicates that improving sleep quality may lead to a reduction in substance abuse during follow-up^20^,^21^. Although an association between opioid dependence and sleep quality has been identified in several studies^22^,^23^, it remains unclear whether NMPOU can influence sleep quality or vice versa and whether sleep improvement could be a potential intervention for NMPOU reduction among adolescents. To begin to address these questions, new studies exploring the relationship between NMPOU and sleep quality are needed. To this end, we conducted a large-scale cross-sectional study in Chongqing, China. The two main purposes of our research were as follows:

1. To examine the prevalence of lifetime, past-year and past-month NMPOU and poor sleep in the sample.
2. To explore the relationship between past-month NMPOU and past-month sleep quality after controlling for potential confounders.

## Results

In total, 20,714 students were invited to participate, and questionnaires from 18,686 (90.6%) students were completed and eligible for our study. Table 1 provides the basic demographic information for the total sample and subgroups with poor sleep or past-month NMPOU. Among the final sample, 18,686 high school students were included, and the proportion of girls was 52.5%. The students ranged in age from 11 to 20 years old, and the mean age was 15.43 (±1.76) years; 41.6% were junior high school students, and 58.4% were senior high school or vocational high school students. Approximately 54.9% of the students lived with both biological parents, and 10.5% reported their family economic status as above average. As for school-related factors, 13.6% of the participants thought their academic pressures were above average, whereas 32.2% of the students reported their academic achievement as below average. Regarding personal habits, 8.7% of the students smoked, and 23.3% drank alcohol. Among the students, 15.5% exercised 5–7 days in the past week. Regarding psychosocial characteristics, 9.1% of the students could be classified as having depressive symptoms, 11.0% felt lonely more than 4 days per week, and 2.2% often thought about suicide.

The prevalences of lifetime, past-year and past-month NMPOU were 14.6, 4.6 and 2.8% in the entire sample, respectively. These prevalences and the past-month NMPOU values among subgroups with or without poor sleep are shown in Table 2. Among the total sample, the most commonly used medicine was licorice tablets with morphine (9.1, 2.5 and 1.5% for lifetime, past-year and past-month, respectively), followed by cough syrup with codeine, Percocet, diphenoxylate and tramadol. Generally, the prevalences of any and specific past-month NMPOU was higher among subgroup with poor sleep based on the Chi-square tests.

Of the total sample, 18.0% were classified as poor sleepers (27.4% of the subjects with past-month NMPOU). The mean CPSQI global score in the total sample was 5.24 (±2.62) points. The participants who indicated past-month NMPOU had a mean global score of 5.97 (±3.24) points.

Based on Chi-square tests, past-month NMPOU was significantly correlated with gender, age, grade, academic achievement, smoking habits, drinking habits, having depressive symptoms, feelling lonely and suicide ideation. These results are shown in Table 1.

Without adjustment for other variables, having poor sleep was significantly correlated with past-month NMPOU (OR = 2.09, 95% CI 1.71 to 2.56), gender, age, grade, living arrangement, family economic status, academic pressure, academic achievement, smoking habits, drinking habits, exercise, having depressive symptoms, feeling lonely and suicide ideation. These results are shown in Table 3.

The final multilevel logistic regression that explore the association between past-month NMPOU and poor sleep is presented in Table 3. After adjusting for potential confounders (gender, age, grade, academic achievement, smoking habits, drinking habits, having depressive symptoms, feeling lonely and suicide ideation), the association between past-month NMPOU and poor sleep remained significant (AOR = 1.47, 95% CI 1.17 to 1.85).

Repeated analyses were conducted to investigate the association between lifetime and past-year NMPOU and poor sleep. After adjusting for potential confounders, the association between lifetime and past-year NMPOU and poor sleep remained significant (AOR = 1.43, 95% CI 1.28 to 1.60; and AOR = 1.68, 95% CI 1.41 to 2.00, respectively) (data not shown in Table).

## Discussion

NMPOU is a growing public concern, and many studies focused on this problem have been conducted^24^,^25^,^26^. In this study, the prevalences of lifetime, past-year and past-month NMPOU were 14.6, 4.6 and 2.8% among the participants, respectively. The most commonly used medicine was licorice tablets with morphine (9.1, 2.5 and 1.5% for lifetime, past-year and past-month nonmedical use, respectively), followed by cough syrup with codeine, Percocet, diphenoxylate and tramadol. The prevalence of past-year NMPOU was lower than that described in a Canadian survey, which reported that 6.6% of the participants (aged 15–17) had engaged in past-year NMPOU^27^. A possible explanation for the discrepancy in the prevalence could be the differences in the classes of medicines considered in these two studies. The Canadian survey included all pain relievers, whereas our study included only five specific categories of medicine. The prevalence of past-month NMPOU was slightly higher than that described in the latest report from the 2014 National Survey on Drug Use and Health (NSDUH), which found that 1.9% of adolescents (aged 12–17) had used pain relievers nonmedically during the past month^28^. Differences in the study samples may contribute to part of the variance in the prevalence. The NSDUH is a national representative survey of the civilian, noninstitutionalized population (aged 12 years or older) of the United States; therefore, it reveals the average prevalence of NMPOU among the entire country. However, our study was conducted only in Chongqing, a single city in central China. Moreover, Shield’s research has indicated that inconsistent questions assessing the presence of NMPOU in different studies could result in large variation^29^.

Regarding sleep quality, 18.0% of the students in this study could be classified as poor sleepers (27.4% among the subgroup with past-month NMPOU). The prevalence of poor sleep was slightly lower than that reported in a study conducted in northern China, which found that 16.9% of the adolescents suffered sleep problems^30^. This result indicates that although sleep disturbance was not rare among the study sample, it was particularly high among the subgroup with past-month NMPOU, meriting further consideration.

To the best of our knowledge, the current investigation is the first study to examine the relationship between past-month NMPOU and sleep quality among Chinese high school students. In this study, we found that the prevalences of any and specific past-month NMPOU were generally higher among subgroups with poor sleep than those without poor sleep. The association between past-month NMPOU and poor sleep was significant after adjusting for gender, age, grade, academic achievement, smoking habits, drinking habits, the presence of depressive symptoms, loneliness and suicide ideation. Previous studies have shown that health risk behaviors (e.g., smoking, alcohol drinking, substance use, etc.) tend to cluster together^31^,^32^. Our research further supported that theory: among participants with past-month NMPOU, the prevalences of smoking and drinking were significantly higher than those in non-users. Although we did not assess the participants’ knowledge and attitudes towards sleep hygiene, given the clustering of the risk behaviors mentioned above and the fact that the most common motivations for nonmedical opioid users are to “experiment or relax or get high”^33^, it is possible that participants with NMPOU also had bad sleep habits and thus suffered poor sleep^34^.

Recent evidence also suggests a biological plausibility of the relationship between NMPOU and poor sleep. There are two speculations of the mechanism of this association. First, opioid use can induce the reduction of adenosine in sleep-regulating brain regions (the pontine reticular formation and substantia innominata region of the basal forebrain)^35^, and adenosine has long been known as a promoter of sleep^36^. Furthermore, prior studies have also shown that by agitating μ-opioid receptors, opioids can decrease GABAergic transmission in the oral part of the pontine reticular nucleus, which regulates sleep and wakefulness, thereby disrupting sleep^37^,^38^.

Based on data from the National Survey on Drug Use and Health, Mark and colleagues found that a major depressive episode is an important risk factor for NMPOU among adolescents^39^. Sleep problems have also been strongly associated with depression in prior studies^40^, potentially explaining the association between past-month NMPOU and poor sleep from a different perspective. Overall, it is clear that NMPOU and poor sleep are correlated with each other, and programs to decrease NMPOU should also pay attention to sleep hygiene and vice versa. Longitudinal studies are also needed to further examine the causal relationship between these two variables.

The strengths of the current study include the large epidemiological sample, the number of control variables and the use of well-validated measures to examine sleep quality. More importantly, this study is the first to investigate the association between past-month NMPOU and sleep quality among high school students. However, this study is subject to several limitations that are common to survey studies. First, because of the cross-sectional design, the cause-effect relationships between past-month NMPOU and sleep could not be determined. We cannot draw conclusions about causality, and longitudinal studies are necessary in the future. Second, because the data were based on high school students, the findings may not be generalizable to younger populations, out-of-school adolescents or older populations. Moreover, NMPOU was narrowly defined in this study because we included only the most frequently used drugs; therefore, the association between past-month NMPOU and sleep may not be generalizable to other opioid drugs. Finally, our research was based on self-report surveys in a school setting, and we thus cannot completely eliminate the possibility of recall bias and reporting bias.

In conclusion, the current study indicates that NMPOU and poor sleep are not rare among Chinese high school students; thus, this top warrants more attention from policymakers, schools and families, as well as further studies on these issues. We also found a significant association between past-month NMPOU and poor sleep, wherein past-month NMPOU was more prevalent among participants with poor sleep than those without poor sleep. Additionally, students who engaged in NMPOU during the past month reported a higher prevalence of poor sleep than non-users. The correlations between these two variables suggest that preventive and intervention programs to decrease NMPOU or poor sleep among adolescents should consider the influence of the other factor.

## Methods

### Participants

A cross-sectional study was conducted to investigate the prevalence of past month NMPOU and to examine its relationship with sleep quality. The participants were high school students from Chongqing, China. Multistage stratified cluster sampling was used to randomly sample high school students from schools in Chongqing. In stage 1, based on GDP level (i.e., more developed district, moderately developed district and less developed district), we randomly selected two counties from each of the three district types. In stage 2, schools in each county were selected. The schools were divided into the following three categories: junior high schools (grades 7–9), senior high schools (grades 10–12) and vocational high schools (grades 10–12). Six junior high schools, four senior high schools and two vocational high schools were randomly selected from each county. In stage 3, two classes were randomly selected from each grade at the selected schools, and all available students in the classes were surveyed. To avoid any potential information bias, students were asked to complete the anonymous questionnaires with the supervision of research assistants while their teachers were absent. In total, 20,714 students were invited to participate, and questionnaires from 18,686 (90.6%) students were completed and qualified for our study. The students’ ages ranged from 11 to 20 in the final sample, and girls comprised 52.5% of the sample. All data were collected between March 2012 and May 2012. This study was approved by the Institutional Review Board of the Sun Yat-sen University School of Public Health. All participants were fully informed of the purpose of the study and were invited to voluntarily participate. Written informed consent letters were obtained from the schools, each participant and one parent of each participant. The methods were carried out in accordance with the approved guidelines.

### Measures

#### Sociodemographic characteristics

Demographic variables: Data were collected on the participants’ gender, age, and grade.

Family-related and school-related factors: Living arrangements were assessed by asking who lived in their primary home. Family economic status was measured by asking about the student’s perception of his or her family’s current economic status (responses were coded as ‘above average’, ‘average’ and ‘below average’). Brothers and sisters were assessed by asking the number of blood brothers and sisters (responses were coded as ‘none’ and ‘≥1’). Academic pressure and academic achievement were measured by asking about the student’s personal appraisal of academic stress or achievement relative to that of his or her classmates (responses were coded as ‘above average’, ‘average’ and ‘below average’).

Personal habits: Smoking habits were assessed by asking the following question: “Have you smoked one or more cigarettes at least one day during the past month?” The response options were either (1) “Yes” or (2) “No”. Drinking habits were assessed by asking the following question: “Have you drunk at least one glass of alcohol (a glass of alcohol is equivalent to a half bottle or can of beer, a small cup of white spirits, or a glass of wine or rice wine) at least one day during the past month?” The response options were either (1) “Yes” or (2) “No”. Physical habits were assessed by asking the following question: “In the past week, how many days have you exercised longer than 30 minutes (sports such as running, swimming, and basketball, or similar activities)?” The response options were (1) “0 days”, (2) “1–2 days”, (3) “3–4 days”, or (4) “5–7 days”.

Psychosocial factors: Loneliness was assessed by asking the following questions: “During the past 12 months, how often did you feel lonely per week?” The response options ranged from 1 (never) to 4 (more than 4 days). Suicide ideation was assessed by asking the following question: “During the past 12 months, have you ever thought about suicide?” The response options were (1) “never”, (2) “occasionally (1–2 times)”, (3) “sometimes (3–6 times)”, and (4) “often (more than 6 times)”. Depressive symptoms were assessed using the *Center for Epidemiology Scale for Depression (CES-D).* The participants were asked to rate the frequency of 20 depressive symptoms over the past week by choosing 1 of 4 response options ranging from “rarely or none of the time” to “most or all of the time”^41^. The scores ranged from 0 to 60. We adopted the 80^th^ percentile as the cut-off (i.e., a score greater than 28 indicated depressive symptoms), and the area under the ROC curve was 0.78.

### NMPOU

Lifetime NMPOU was assessed by the following question: “Have you ever used the following medications, even once, when you were not sick or just for the intended purpose to experiment or to get high without a doctor’s prescription?” The question was followed by a list of prescription opioid drugs. The response options were “Yes” and “No”. If the answer was yes, we then asked about the participant’s past-year NMPOU, and students who reported NMPOU during the past year were further asked about the past-month NMPOU. In this study, we included only five opioid drugs: licorice tablets with morphine, cough syrup with codeine, Percocet, diphenoxylate and tramadol. Lifetime, past-year and past-month NMPOU prevalences were calculated for any use (i.e., use of at least one category at least once) and then separately for each of the five drug categories (the variables are not mutually exclusive). The list of medications was developed based on a list provided by the Center for ADR Monitoring of Chongqing and medicines reported to be widely used by adolescent drug abusers in local rehabilitation centers.

### Sleep quality

The Chinese Pittsburgh Sleep Quality Index (CPSQI) was used to assess sleep quality over a 1-month time interval. The global CPSQI score ranges from 0 to 21, with higher scores indicating worse sleep quality^42^,^43^. It contains seven subscales (subjective sleep quality, sleep latency, sleep duration, habitual sleep efficiency, sleep disturbance, use of sleep medications and daytime dysfunction), and the score for each subscale ranges from 0 to 3 points. In China, a PSQI global score greater than 7 points indicates poor sleep^44^, although an original cut-off of 5 points was recommended by the scale developers^43^. The Chinese version of the PSQI has been demonstrated to be valid and reliable in the Chinese population^44^.

### Statistical analysis

Two investigators independently entered all data using EpiData 3.1. All statistical analyses were conducted using SPSS V.20.0 and SAS V.9.2. Descriptive analyses were used to describe the sociodemographic characteristics for the entire sample and for subgroups with poor sleep or with past-month NMPOU. Chi-square tests were used to test differences between the categorical variables. When the participants were grouped by schools, the ICC (intra-class correlation coefficient) was 0.11, indicating the existence of a group effect. Therefore, multilevel analyses (generalized linear mixed effects models adopting the GLMMIX procedure in SAS) were used in which the schools were treated as clusters. The presence of poor sleep was considered the dependent variable, and past-month NMPOU (i.e., use of at least one category at least once during the past month) was the predictor variable. Covariates that were associated (p value less than 0.05) with both past-month NMPOU and poor sleep, according to the univariate analyses or based on significant evidence in the literature, were entered as confounders in the final regression analysis. Repeated analyses were conducted between lifetime and past-year NMPOU (i.e., the use of at least one category at least once during lifetime or during the past year) and poor sleep. All statistical tests were two-sided, and a p value less than 0.05 was considered statistically significant.

## Additional Information

**How to cite this article**: Tang, D. *et al*. The prevalences of and association between nonmedical prescription opioid use and poor sleep among Chinese high school students. *Sci. Rep.* **6**, 30411; doi: 10.1038/srep30411 (2016).

## Figures and Tables

**Table 1:** Basic demographic information for the sample (N = 18686).

Variable	Total (N = 18686)		With poor sleep (n = 3361)		With past-month NMPOU (n = 519)		p value*
	n	%	n	%	n	%	
Gender							0.03
Girls	9803	52.5	1943	57.8	248	47.8	
Boys	8883	47.5	1418	42.2	271	52.2	
Age (years)							<0.001
11–13	3371	18.0	339	10.1	126	24.3	
14–16	9196	49.2	1676	49.9	255	49.1	
17–20	6119	32.8	1346	40.0	138	26.6	
Grade							<0.001
7th-9th	7771	41.6	992	29.5	270	52.0	
10th-12th	10915	58.4	2369	70.5	249	48.0	
Living arrangement							0.22
Two biological parents	10256	54.9	1743	51.9	266	51.3	
Only father or mother	3101	16.6	574	17.1	96	18.5	
Others	5329	28.5	1044	31.0	157	30.2	
Family economic status							0.05
Above average	1970	10.5	268	8.0	66	12.7	
Average	10760	57.6	1786	53.1	273	52.6	
Below average	5956	31.9	1307	38.9	180	34.7	
Brothers and sisters							0.81
None	6790	36.3	1173	34.9	186	35.8	
≥1	11896	63.7	2188	65.1	333	64.2	
Academic pressure							0.98
Above average	2536	13.6	323	9.6	70	13.5	
Average	8209	43.9	1014	30.2	230	44.3	
Below average	7941	42.5	2024	60.2	219	42.2	
Academic achievement							<0.001
Above average	5805	31.1	852	25.4	117	22.6	
Average	6863	36.7	1218	36.2	174	33.5	
Below average	6018	32.2	1291	38.4	228	43.9	
Smoking habits							<0.001
No	17053	91.3	2901	86.3	426	82.1	
Yes	1633	8.7	460	13.7	93	17.9	
Drinking habits							<0.001
No	14341	76.7	2236	66.5	337	64.9	
Yes	4345	23.3	1125	33.5	182	35.1	
Exercise							0.22
0 days	4283	22.9	1129	33.6	121	23.3	
1–2 days	8029	43.0	1303	38.8	230	44.3	
3–4 days	3475	18.6	485	14.4	79	15.2	
5–7 days	2899	15.5	444	13.2	89	17.2	
Depressive symptoms							<0.001
No	16994	90.9	2404	71.5	433	83.4	
Yes	1692	9.1	957	28.5	86	16.6	
Loneliness							<0.001
Less than 1 day/week	9716	52.0	1025	30.5	209	40.3	
1 to 4 days/week	6917	37.0	1564	46.5	233	44.9	
More than 4 days/week	2053	11.0	772	23.0	77	14.8	
Suicide ideation							<0.001
Never	14669	78.5	2026	60.3	338	65.1	
Occasionally (1–2 times/year)	3062	16.4	895	26.6	115	22.2	
Sometimes (3–6 times/year)	539	2.9	215	6.4	27	5.2	
Often (>6 times/year)	416	2.2	225	6.7	39	7.5	

NMPOU, nonmedical prescription opioid use.

^*^Chi-square tests were used to test the association between the above-mentioned categories and NMPOU status.

**Table 2:** Prevalence of adolescent use of any and specific opioid drugs (N = 18686).

NMPOU					Past-month NMPOU					
	Lifetime NMPOU (N = 18686)		Past-year NMPOU (N = 18686)		Total (N = 18686)		Without poor sleep (n = 15325)		With poor sleep (n = 3361)	
	n	%	n	%	n	%	n	%	n	%
Any NMPOU*	2719	14.6	854	4.6	519	2.8	377	2.5	142	4.2
Licorice tablets with morphine*	1705	9.1	467	2.5	283	1.5	201	1.3	82	2.4
Cough syrup with codeine*	1324	7.1	412	2.2	244	1.3	173	1.1	71	2.1
Percocet*	1010	5.4	329	1.8	205	1.1	135	0.9	70	2.1
Diphenoxylate*	198	1.1	69	0.4	59	0.3	35	0.2	24	0.7
Tramadol*	134	0.7	75	0.4	46	0.2	27	0.2	19	0.6

Note: Chi-square tests were used to compare the prevalence of past-month NMPOU between subjects with and without poor sleep.

*p<0.001

NMPOU, nonmedical prescription opioid use.

**Table 3:** Crude and adjusted odds ratios and 95% confidence intervals of poor sleep among adolescents.

Variables	OR	95% CI	AOR	95% CI
Past-month NMPOU				
No	1.00		1.00	
Yes	2.09	**1.71–2.56**	1.47	**1.17–1.85**
Gender				
Girls	1.00			
Boys	0.83	**0.77–0.90**		
Age (years)				
11–13	1.00			
14–16	1.55	**1.35–1.79**		
17–20	1.66	**1.40–1.96**		
Grade				
7th-9th	1.00			
10th-12th	1.87	**1.42–2.46**		
Living arrangement				
Two biological parents	1.00			
Only father or mother	1.14	**1.02–1.27**		
Others	1.16	**1.06–1.27**		
Family economic status				
Above average	1.00			
Average	1.17	**1.01–1.35**		
Below average	1.61	**1.38–1.87**		
Brothers and sisters				
None	1.00			
≥1	1.04	0.95–1.14		
Academic pressure				
Above average	1.00			
Average	0.93	0.81–1.06		
Below average	2.08	**1.82–2.37**		
Academic achievement				
Above average	1.00			
Average	1.18	**1.07–1.30**		
Below average	1.52	**1.38–1.67**		
Smoking habits				
No	1.00			
Yes	2.03	**1.80–2.29**		
Drinking habits				
No				
Yes	1.89	**1.74–2.06**		
Exercise				
0 days	1.00			
1–2 days	0.57	**0.52–0.62**		
3–4 days	0.49	**0.44–0.56**		
5–7 days	0.56	**0.50–0.64**		
Depressive symptoms				
No	1.00			
Yes	8.03	**7.21–8.95**		
Loneliness				
Less than 1 day/week	1.00			
1 to 4 days/week	2.45	**2.25–2.67**		
More than 4 days/week	5.15	**4.60–5.76**		
Suicide ideation				
Never	1.00			
Occasionally (1–2 times/year)	2.71	**2.47–2.98**		
Sometimes (3–6 times/year)	4.54	**3.78–5.46**		
Often (>6 times/year)	8.27	**6.73–10.15**		

Bold type indicates that the CI does not include the null according to logistic regression analyses.

OR, odds ratio by univariate logistic regression; AOR, adjusted odds ratio by multilevel multivariate logistic regression; NMPOU, nonmedical prescription opioid use.
